# Does commuting time affect labour’ diet health?

**DOI:** 10.3389/fpubh.2025.1508951

**Published:** 2025-08-28

**Authors:** Shuang Ding, Gangyi Wang, Jiwei Ma

**Affiliations:** College of Economics and Management, Northeast Agricultural University, Harbin, China

**Keywords:** diet quality, commuting, time poverty, income, laborer

## Abstract

Will long commutes affect the health of workers? Numerous studies have shown that long commutes can lead to a decline in personal health, and the cause of this decline is generally believed to be an increase in stress. This study starts from the perspective of diet, combines income variables, and comprehensively analyzes the positive impact of income on the diet of workers and the negative impact of commuting time on the diet of workers. New evidence has been added to the impact of long commutes on workers’ health. The empirical results based on the CHNS survey data show that: (1) there is an inhibitory effect of commuting time in the process of income contributing to the improvement of Chinese laborers’ diet quality. (2) longer commuting time reduces laborers’ intake of fish, seafood, vegetables, eggs, milk, and nuts, which leads to a decrease in the overall diet quality. (3) longer commuting time has a more negative impact on the quality of laborers’ diets, and this effect is further amplified with age. (4) Increased commuting time leads to a decrease in the dietary quality of laborers’ children. Therefore, there is a need to shorten the commuting time of workers, optimize the urban structure, and promote the formation of a polycentric urban pattern to alleviate the imbalance in the dietary structure of residents.

## 1 Introduction

Long commutes have become a major health threat to laborers. In recent years, during the rapid development of China’s urbanization, laborers’ commuting time has been lengthening, and the prevalence of long commuting hours has made China’s laborers feel more and more time-poor and powerless to dominate their time (1). According to the “2022 China’s Major Cities Commuting Monitoring Report,” more than 14 million people in the country’s major cities suffer from extreme commuting (i.e., one-way commuting time of more than 60 min) (2). With the development of society and economy and the rising income level of the population, absolute economic poverty has been eliminated, and a new kind of poverty has emerged, “time poverty (viewing time as a scarce resource indicates people’s subjective feelings about time scarcity)” (3, 4). According to an actual study of Beijing residents, the average one-way commute time in Beijing is 40 min, which means that laborers need to spend 1 h and 20 min a day on commuting (5). Time compression has led to dramatic changes in the diet of the population. Many laborers tend to eat out, which saves time on commuting home and preparing food. This is especially prevalent among doctors and healthcare professionals. Studies have shown that when commuting more than 6 h per week, the health of laborers decreases, and this decrease in health includes a decrease in physical activity, physical health, and mental health (6–9). Research confirms that a longer commuting time is, moreover, related to more visits to the general practitioner (10). However, there is a lack of direct evidence on the impact of increased commuting time on labor health.

In this study, we start with diet quality to analyze the effect of commuting time on labor health. And take the dual constraints of income and time into account, fully consider the relationship between the dietary quality of laborer’ eating out and the cost of income and time and focus on the impact of the cost of time on the dietary quality of laborers. Using data from the CHNS database, we will empirically test the quality of laborers’ diets under the dual constraints of income and commuting time and looking for direct evidence of the effect of increased commuting time on labor health.

## 2 Theoretical analysis and research methodology

### 2.1 Theoretical analysis and research hypotheses

Household utility maximization. The basic framework of the Becker model (11) is that under the production, income, and time constraints, households maximize household utility by producing different product combinations and consuming different product combinations. According to Becker’s (11) theory of household production and consumption, a household member’s food consumption is not only affected by household income, but also by its own time constraints and those of its family members at the same time. A household produces consumer goods, $z_{i}$(*i = 1,2…n*.), by choosing different combinations of factors and maximizes household utility (U) by consuming these goods. The optimization problem in Becker’s model can be described by the following equation:


(1) $$\max\;U=U(z_{1},z_{2},z_{3},\ldots z_{n},D),$$

(2) $$s.t.z_{i}=z_{i}(x_{i},t_{i1},t_{i2},\ldots t_{\mathrm{im}}),i=1,2,3,\ldots ,m,$$

(3) $$\;T_{k}=T_{\mathrm{lk}}+\sum_{i=1}^{n}t_{\mathrm{ik}},k=1,2,3,\ldots ,n,$$

(4) $$\sum_{k=1}^{m}w_{k}T_{\mathrm{lk}}+v=\sum_{i=1}^{n}p_{i}x_{i},$$

Equation 1 is the household utility maximization achieved by combining different consumer goods, where *D* is the other variable reflecting household heterogeneity; Equation 2 expresses the fact that household production of consumer goods $z_{i}$ is jointly constrained by $x_{i}$ inputs needed for production, time needed for production $t_{\mathrm{ik}}$ and production technology $z_{i}$; Equation 3 describes the time constraint on household production, where $T_{k}\;$ and $T_{\mathrm{lk}}$ represent the total available time of household member k and his labor supply time in the labor market, respectively; Equation 4 is the household’s income constraint, where $w_{k}$ represents the wage of household member k, v represents the household’s non-wage income, and $p_{i}$ represents the price of input $x_{i}$.

Individual utility maximization. According to Becker’s household production function and utility maximization theory, we can assume that work groups are rational decision makers who can make optimal decisions according to their personal preferences to maximize the utility of their own dietary quality in the face of limited leisure time during the working day and overall constrained income. Based on this a model of dietary consumption behavior under income and time constraints can be constructed (see Equation 5):

Assume that the individual utility function is:


(5) $$U=U(Q_{f},Q_{o},L),$$

Where $Q_{f}\;$ denotes the quality of food consumption, $Q_{o}$ denotes consumption of other goods and services, and L denotes leisure time. Consumers face the following budget constraint (see Equation 6):


(6) $$P_{f}Q_{f}+P_{o}Q_{o}=\mathrm{wH}=I,$$

Where $P_{f}$ is the price of food, $P_{o}$ is the price of other goods, I is total income, w is the wage rate, and H is the number of hours worked. Meanwhile, consumers face time constraints (see Equation 7):


(7) $$H+L+T_{f}=T,$$

Where $T_{f}$ denotes the food-related time investment (e.g., food procurement, meal preparation, etc.) and T is the total time.

Income effects in diet quality. The traditional view is that income determines the quantity and quality of residents’ food consumption, and income growth is an important way to optimize residents’ dietary structure and alleviate malnutrition (12–14). Research shows that the food expenditure of low-income groups is mainly based on grains consumption, along with this rise in income food consumption is gradually more nutritious, healthier, the residents of nutrition to achieve a balanced intake. In other words, when the income of the working group I increased, will bring about an increase in food consumption $Q_{f}$.

In summary, Hypothesis 1 is proposed.

H1: Higher income significantly improves individual dietary quality.

Time constraint effect. Time constraints refer to the restricted allocation of an individual’s time among activities and corresponding subjective states during a given period (15). Time use studies were an important element of early sociology used to evaluate the labor burden of the working class (11). In recent years, Internet buzzwords such as “996 work system” (going to work at 9 a.m. and getting off at 9 p.m., working 6 days a week) and “007 work system” (working from 0:00 to 0:00, working 7 days a week), which reflect the schedule in China, have frequently gained heated debates and reflect this fact that time can be allocated to the laborer. This fact is also reflected in the fact that the reduced availability of time has increased the labor burden of workers. When economic development has raised the income level of the residents, time is constantly compressed, time poverty has become an important factor affecting the lives of our residents. Under the general social phenomenon of rising income and tightening time, the frequency of eating out for laborers has shown an increasing trend (16). Studies have shown that eating out is associated with higher energy and lower dietary fiber, phosphorus, potassium, folic acid, vitamin C and other nutrients (17, 18), which indirectly leads to a decline in the quality of the laborers’ diet. A study on the relationship between eating out and residents’ health among men in China pointed out that residents who eat out have high BMI, waist circumference, diastolic blood pressure, body fat percentage, and TG levels (19). Existing studies have shown that eating out is associated with unfavorable dietary nutrition, but eating out has become the optimal choice under the time squeeze of working groups. On the one hand, reduced leisure time makes consumers lack cooking energy and rely more on takeaway fast food; on the other hand, reduced food procurement time can limit access to quality ingredients. The effect of increased commuting time ($T_{c}$) on diet-related time can be expressed by Equation 8:


(8) $$C(T_{c})=I(T_{c})+s(T_{c})$$

Where, I is the wage rate, s$\left(T_{c}\right)$ is the mealtime cost of commuting to the individual, and s$\left(T_{c}\right)$>0. Since $\frac{\partial c}{\partial T_{c}}=I+s\prime (T_{c})>0$, the longer the commute time, the higher the time cost, consumers will take more time-saving measures, such as choosing take-out, reducing the number of times of cooking, etc., which affects the quality of the individual’s meal.

In summary, Hypothesis 2 is proposed.

H2: Increased commuting time significantly reduces individual diet quality.

Time-value effects. According to the theory of urban spatial equilibrium, variables such as labor wages, commuting costs, and housing prices are endogenous, and laborers make trade-offs between multiple variables. When laborers accept a long commute, they can be compensated accordingly in other ways, such as higher wage income. Stutzer and Frey (20) empirically investigate this compensatory behavior and find that people’s subjective well-being decreases the longer the commute, and refer to this utility imbalance, where a longer commute does not necessarily lead to a corresponding compensation, as a “Commuting Paradox”. Does this “cost–benefit imbalance” exist in objective health? Whether commuting time “consumes” dietary quality while high income “compensates” for dietary quality deserves further discussion. This relationship can be expressed as a function of Equation 9:


(9) $${\frac{\partial^{2}T_{f}}{\partial T_{c}\partial I}|}_{T_{c}↑}<0,$$

In summary, Hypothesis 3 is proposed.

H3: There is a negative moderating effect of commuting time in the effect of income on diet.

### 2.2 Model construction

The model is constructed through the above analysis. Since the dependent variables are dichotomous, this study chose to use Logit models, which are set up as Equation 10:


(10) $${\text{CHEI}}_{i}=\alpha {\text{Commut}}_{i}+\beta {\text{incom}}_{i}+\gamma {\text{Control}}_{i}+\mu_{i},$$

where ${\text{CHEI}}_{i}$ is individual diet quality, ${\text{Commut}}_{i}$ is commuting time, ${\text{incom}}_{i}$ is per capita household income, and ${\text{Control}}_{i}$ is variables such as demographic characteristics and household characteristics that affect diet quality.

## 3 Data and variables

### 3.1 Data sources

The retrospective data used in this study were from the China Household Nutrition and Health Survey (CHNS). The CHNS is an international collaborative project between the University of North Carolina and the Center for Nutrition and Health of the Chinese Center for Disease Control and Prevention (CDC), which began in 1989 and collected data from 15 provinces, autonomous regions, and municipalities in eastern, middle, and western China, including 220 community samples, 7,200 household samples, and 30,000 resident samples, which is a good national representation.

As needed, this study utilized CHNS data to filter out those who responded to the questionnaire on commuting time from those aged 25 years (including 25) (fully educated) to (60 years including 60) (retirement age). Subsequently, the diet quality scores of the samples were calculated, based on the results, the samples with too low scores (below 15 points) were deleted. After matching the control variables, the sample with household per capita income of 0 was deleted. After deleting the missing values that were not answered in the questionnaire, 2,567 sample data were obtained.

### 3.2 Variable description

#### 3.2.1 Dependent variable

In this study, the Chinese Healthy Eating Index (CHEI) (21) was used to measure the dietary quality of the population. The CHEI used the standard portion (SP) food division method proposed by the DGC-2016 to ensure that there was a consistent amount of energy, carbohydrates, fats, and proteins in each food group. This evaluation method eliminates the ambiguity associated with existing evaluation methods (BDI, CFPS scores) that evaluate nutrient intake based on absolute weight [grams (g) or kilograms (kg)].

The CHEI scoring system was divided into 17 food groups, of which 12 assessed dietary adequacy (the “moderate” intake category recommended in the Dietary Guidelines) and 5 assessed dietary restriction (the “limited” intake category recommended in the Dietary Guidelines). All food components in the 17 food groups were energy-adjusted by density (per 1,000 calories) except for added sugars (percentage of energy) and alcohol (absolute intake), which were scored as shown in the table below. The intake between 0 and the recommended value will be calculated according to the following formula:


Score=(actual intake/recommended intake)×full score

Therefore, we first categorized the foods in the dietary survey data according to the 17 food groups in the CHEI, and then converted the daily intake of g for each food into SP, and then summed up the SP of the corresponding foods by food group, divided it by the average energy intake of the day, and finally scored it according to the criteria. The scoring criteria are shown in Tables 1 and 2.

**Table 1:** Composition and scoring standards of CHEI dietary quality indicators.

Food group	Score			
	0		5	10
Adequacy				
Total grains	0	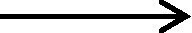	≧2.5SP/1000 kcal	
Whole grains and mixed beans	0	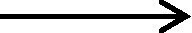	≧0.6SP/1000 kcal	
Tubers	0	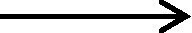	≧0.3SP/1000 kcal	
Total vegetables	0	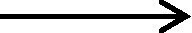	≧1.9SP/1000 kcal	
Dark vegetables	0	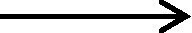	≧0.9SP/1000 kcal	
Fruits	0	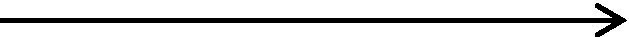		≧1.1SP/1000 kcal
Dairy	0	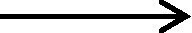	≧0.5SP/1000 kcal	
Soybeans	0	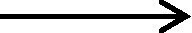	≧0.4SP/1000 kcal	
Fish and seafood	0	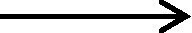	≧0.6SP/1000 kcal	
Poultry	0	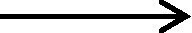	≧0.3SP/1000 kcal	
Eggs	0	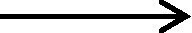	≧0.5SP/1,000 kcal	
Seeds and nuts	0	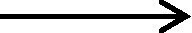	≧0.4SP/1,000 kcal	
Limitation				
Red meat	≧3.5	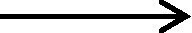	≦0.4SP/1,000 kcal	
Cooking oils	≧32.6	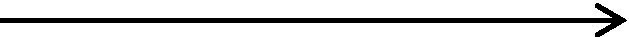		≦15.6 g/1,000 kcal
Sodium	≧3608	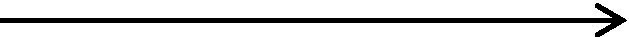		≦1,000 mg/1,000 kcal
Added sugars	≧20%	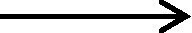	≦10% of energy	
Alcohol	≧25 g (men)/15 g (woman)	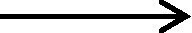	≦60 g (men)/40 g (women)	

**Table 2:** CHEI ingredient and scoring criteria comparison table.

Food group	The standard portion per 1,000 kcal										
Calorie level (kcal)	≤1,000	≤1200	≤1400	≤1600	≤1800	≤2000	≤2200	≤2400	≤2600	≤2800	≤3000
Total grains	1.7	1.7	2.1	2.5	2.5	2.5	2.5	2.5	2.7	2.7	2.7
Whole grains and mixed beans	/	/	/	0.6	0.8	1.0	1.1	1.3	/	/	/
Tubers	/	/	/	0.3	0.3	0.4	0.3	0.4	0.5	0.4	0.4
Total vegetables	2.0	2.1	2.1	1.9	2.2	2.3	2.0	2.1	1.9	1.8	2.0
Dark vegetables	1.0	1.0	1.1	0.9	1.1	1.1	1.0	1.0	1.0	0.9	1.0
Fruits	1.5	1.3	1.1	1.3	1.1	1.5	1.4	1.5	1.3	1.4	1.3
Dairy	2.0	1.7	1.0	0.8	0.7	0.6	0.5	0.5	0.5	0.4	0.4
Soybeans	0.3	0.6	0.5	0.5	0.4	0.4	0.6	0.5	0.5	0.4	0.4
Seeds and nuts	/	/	/	0.6	0.6	0.5	0.5	0.4	0.4	0.4	0.3
Fish and seafood	0.3	0.4	0.6	0.6	0.6	0.6	0.8	0.7	0.6	0.8	0.9
Poultry	0.3	0.5	0.6	0.6	0.6	0.6	0.8	0.7	0.6	0.8	0.7
Eggs	0.4	0.5	0.4	0.6	0.5	0.6	0.5	0.5	0.4	0.4	0.4
Cooking oils (g/1000 kcal)	15.0	16.7	14.3	15.6	13.9	12.5	11.4	12.5	11.5	10.7	11.7

The recommended portion size of the food group is expressed in standard portions per 1,000 kilocalories (SP).

#### 3.2.2 Independent variable

Commuting time and income. Commuting time: This study uses three modes of commuting including walking, public transportation, and bicycling entered in the CHNS database, and counts the commuting time (in h) of the 25–60-year-old working group who use different modes for commuting. Income: Considering that individual income receives multiple influences from the number and structure of family members as well as the income of other members of the family, per capita household income was chosen as the independent variable in this study (see Table 3).

**Table 3:** Descriptive statistics of variables are cited.

Variable classification	Variable	Obs	Mean	Std. dev.	Min	Max
Dependent variable	CHEI (sample)	2,567	61.9064	10.0720	16.9785	95.7153
	CHEI (sample’s children)	338	59.8878	11.5338	19.7980	87.9166
	CHEI (sample’s parents)	471	61.1472	9.5900	35.4666	93.2796
Independent variable	Income (ln)	2,567	6.9559	0.8808	4.6068	9.9200
	Commuting time (h)	2,567	0.6084	0.6791	0.0500	5
	Meals at corporation (frequency)	2,567	0.7534	1.4389	0	9
	Meals at restaurant (frequency)	2,567	0.9384	1.5994	0	9
	Meals at home (frequency)	2,567	7.1246	2.2686	0	9
	Total of meals eaten out (frequency)	2,567	1.8753	2.2686	0	9
Control variables	BMI	2,567	23.6625	3.7392	12.1245	58.2333
	Education level	2,567	3.1208	1.4871	0	6
	Knowledge score	2,567	36.6506	5.4991	0	54
	Marital status	2,567	2.0721	0.6798	0	9
	Mall score	2,567	6.7604	2.6684	0	10
	Transportation score	2,567	6.2122	1.9651	0	10
Instrumental variable	Bathroom area (m^2^)	2,567	3.6276	3.5987	0	30
	Average price of commercial housing in each province (ln)	2,567	8.2289	0.7305	7.0745	9.6497

Data source: statistical analysis based on data from China Health and Nutrition Survey in 2004, 2006, 2009 and 2011. Instrumental variables were obtained from the publicly available data of the National Bureau of Statistics of China (https://www.stats.gov.cn/index.html).

#### 3.2.3 Control variable

This paper mainly analyzes the effects of commuting time and income on dietary quality, to exclude the interference of other factors on the empirical results, and more accurately assess the effects of commuting time and income on dietary quality, this study will add control variables from the following aspects.

According to previous research results, individual’s education level, physical condition and marital status can all have an impact on dietary quality. In addition, the level of shopping malls in the place of residence also determines whether individuals can obtain enough abundant food. The traffic conditions of the place of residence also determine that individuals are able to buy food conveniently. Therefore, education level and dietary knowledge are selected for demographic characteristics; marital status is selected for family variable characteristics; BMI is selected for health characteristics; and mall scores and transportation scores are used as control variables in consideration of the availability of eating out. Some variables had a small number of missing values, which were deleted without affecting the overall estimation, and the main variables are shown in Table 3. And Stata MP 17 was used for empirical analysis.

#### 3.2.4 Statistical analysis of diet quality in the working population

Diet quality is an assessment of the health status of dietary patterns aimed at lasting improvements in individual and overall health (22). Studies have found that poor dietary quality is prevalent in the Chinese population. Unhealthy dietary structure may lead to malnutrition decreased physical functioning and susceptibility to chronic diseases in the population.

The distribution of CHEI scores by province for the Chinese working population (25–60 years old) is shown in Table 4. The dietary quality scores of Chinas working population are generally low, with 44.56% of workers having dietary quality scores lower than 60, and only 21.23 of workers having dietary quality in a superior state. The overall distribution of CHEI scores is mainly concentrated in the range of 45–60 points, and only 0.16% of the workers’ diet quality reaches more than 90 points. Moreover, the number of workers with poor dietary quality is related to the level of regional economic development, and the dietary quality of workers in regions with poor levels of regional economic development is even lower.

**Table 4:** Distribution of dietary quality (CHEI) of residents in each province (%).

Province	Dietary quality distribution %					
	Dietary defect			Dietary balance		
	High	Moderate	Low	High	Moderate	Low
	45 < =CHEI	45 < CHEI<=60	60 < CHEI<=70	70 < CHEI<=80	80 < CHEI<=90	90 > CHEI
Beijing	0.0390	1.2855	2.6490	3.7008	0.8960	0.0779
Shanghai	0.0390	1.9868	3.7008	2.9217	0.5064	0
Jiangsu	0.0390	2.9217	3.7398	1.8699	0.5843	0.0779
Liaoning	0.1169	1.8699	3.7008	2.9996	0.3506	0
Chongqing	0.2727	2.5321	1.3245	0.3116	0	0
Heilongjiang	0.2727	2.9996	4.3241	3.6229	0.7791	0
Henan	0.3116	3.2333	2.0257	0.5454	0	0
Hubei	0.3116	3.1165	2.8048	0.3896	0	0
Hunan	0.3116	5.4149	3.1944	1.0129	0	0
Shandong	0.3506	4.7916	2.9607	0.4675	0	0
Guizhou	0.5064	6.9342	2.7659	0.1169	0	0
Guangxi	1.4414	3.4671	1.0518	0	0	0

Data source: statistical analysis based on the China Health and Nutrition Survey data in 2004, 2006, 2009 and 2011.

**Figure 1:**
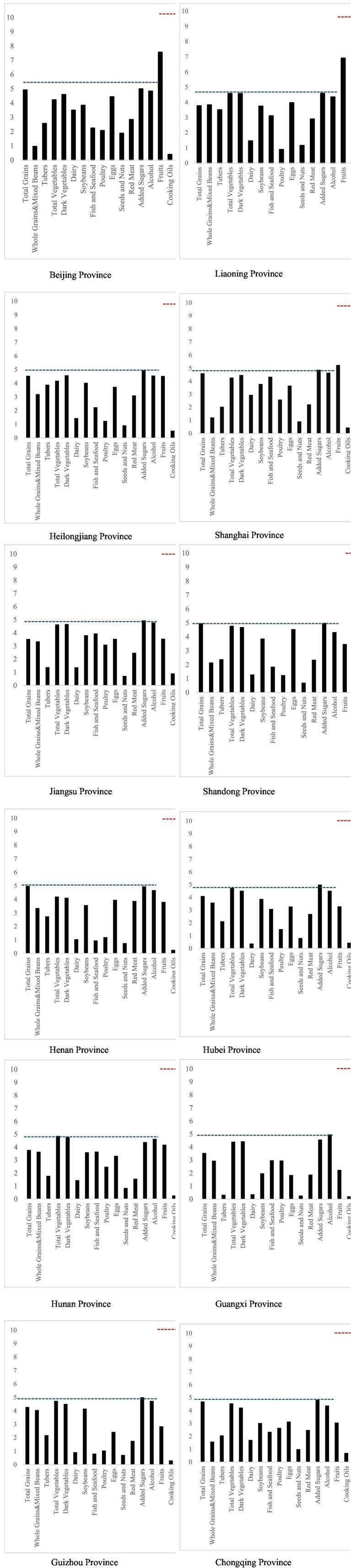
Distribution of provinces in each index segment of CHEI. Data source: statistical analysis based on the China Health and Nutrition Survey data in 2004, 2006, 2009 and 2011.

Distribution of provinces in each index segment of CHEI is shown in Figure 1. From the overall distribution, the insufficient intake of residents’ dietary quality is mainly concentrated in meat, eggs, milk, nuts, edible oils, and Whole Grains and Mixed Beans in some areas, Fish and Seafood in some areas, which is related to the geographic location and dietary habits of the provinces. According to the data of each province, there is regional heterogeneity in the dietary imbalance status of the population. The blue and red dotted lines in the picture are standardized food intake scores.

## 4 Empirical results

### 4.1 Benchmark regression analysis

#### 4.1.1 Income and dietary quality

Table 5 reports the results of the regression of income on diet quality. When no other variables are controlled for, the higher the income the higher the quality of the resident’s diet. The coefficient of income on the quality of the resident’s diet decreases with the gradual addition of the control variables. As shown in Table 5 (5), the regression coefficients of income on dietary quality become larger after controlling for commuting time, proving that there is a time constraint in the effect of income on dietary quality. Commuting time reduces the positive effect of income on diet quality and is negatively related to diet quality. To further support this result, further regression analyses of commuting time and diet quality will be conducted. Hypothesis H1 is tested.

**Table 5:** Regression results of income and diet quality.

Variable	(1)	(2)	(3)	(4)	(5)
	CHEI	CHEI	CHEI	CHEI	CHEI
Income	3.4300***	2.6309***	2.6424***	1.1890***	1.1755***
	(0.0000)	(0.0000)	(0.0000)	(0.0000)	(0.0000)
BMI		−0.0393	−0.0411	−0.1077**	−0.1083**
		(0.4362)	(0.4157)	(0.0188)	(0.0179)
Education level		0.9236***	0.9070***	0.5232***	0.5396***
		(0.0000)	(0.0000)	(0.0001)	(0.0001)
Knowledge score		0.0879**	0.0899***	0.0364	0.0356
		(0.0111)	(0.0095)	(0.3082)	(0.3181)
Marital status		0.1199	0.1122	0.3061	0.2659
		(0.6653)	(0.6856)	(0.2186)	(0.2842)
Mall score			−0.0904	0.0273	0.0230
			(0.2083)	(0.6783)	(0.7256)
Transportation score			0.1440	−0.0032	0.0048
			(0.1424)	(0.9736)	(0.9595)
Commuting time					−1.0387***
					(0.0000)
Constant	38.0480**	38.1835***	37.8570***	60.2524***	61.5167***
	(0.0000)	(0.0000)	(0.0000)	(0.0000)	(0.0000)
Control time effect	Yes	Yes	Yes	Yes	Yes
Control area effect	Yes	Yes	Yes	Yes	Yes
*N*	2,567	2,567	2,567	2,567	2,567
*R* ^2^	0.0896	0.1049	0.1054	0.2905	0.2949

*, ** and *** indicate significant at the 10, 5 and 1% levels, respectively. *p*-Values in parentheses.

#### 4.1.2 Commuting time and dietary quality

Table 6 reports the results of the regressions of commuting time on dietary quality. In all four sets of regression results, there is a significant negative correlation between commuting time and the quality of laborers’ diets. This indicates that an increase in commuting time does reduce the quality of laborers’ diets. Among other variables affecting residents’ dietary quality, dietary knowledge and education level are positively associated with dietary quality. Meanwhile, the higher the mall score and the better the transportation condition, the higher the residents’ dietary quality score. Hypothesis H2 was tested.

**Table 6:** Regression results of commuting time and diet quality.

Variable	(1)	(2)	(3)	(4)	(5)
	CHEI	CHEI	CHEI	CHEI	CHEI
Commuting time	−1.0161***	−1.0546***	−1.0543***	−1.0387***	
	(0.0001)	(0.0000)	(0.0000)	(0.0000)	
Commuting time × income					−0.1123***
					(0.0018)
BMI		−0.1043**	−0.1038**	−0.1083**	−0.1034**
		(0.0228)	(0.0237)	(0.0179)	(0.0244)
Education level		0.7849***	0.7831***	0.5396***	0.7973***
		(0.0000)	(0.0000)	(0.0001)	(0.0000)
Knowledge score		0.0396	0.0394	0.0356	0.0396
		(0.2682)	(0.2706)	(0.3181)	(0.2690)
Marital status		0.2394	0.2384	0.2659	0.2425
		(0.3363)	(0.3387)	(0.2842)	(0.3314)
Mall score			0.0140	0.0230	0.0148
			(0.8313)	(0.7256)	(0.8223)
Transportation score			0.0072	0.0048	0.0044
			(0.9397)	(0.9595)	(0.9633)
Income				1.1755***	
				(0.0000)	
Constant	71.2672***	68.7812***	68.6334***	61.5167***	68.3078***
	(0.0000)	(0.0000)	(0.0000)	(0.0000)	(0.0000)
Control time effect	Yes	Yes	Yes	Yes	Yes
Control area effect	Yes	Yes	Yes	Yes	Yes
*N*	2,567	2,567	2,567	2,567	2,567
*R* ^2^	0.2772	0.2896	0.2891	0.2949	0.2870

*, ** and *** indicate significant at the 10, 5 and 1% levels, respectively. p-Values in parentheses.

The interaction term between commuting time and income was added to the empirical model for regression analysis to examine whether commuting time could be able to reduce the improvement in meal quality from income. As shown in Table 6 (5), the coefficient of the interaction term between commuting time and income is negative at the 1% level of significance and the coefficient decreases by 0.9264, commuting time can reduce the enhancement of dietary quality from income.

### 4.2 Robustness analysis

#### 4.2.1 Endogeneity testing

Income and dietary quality. Although the benchmark regression equation controls for individual characteristics and habits, household characteristics, food convenience, food accessibility, region and year as much as possible, addressing endogeneity due to omitted variables, there may still be endogeneity due to bidirectional causality between income and dietary quality. Therefore, this study sets the residential environment instrumental variable (bathroom area of apartment) and uses 2sls regression for endogeneity test. According to the regression results of (1) in Table 7, the coefficient on income is significantly positive at the 1% level after addressing endogeneity, indicating that income and can significantly improve the quality of laborer’ diets.

**Table 7:** Regression results of endogeneity testing.

Variable	(1)	(2)	(3)	(4)	(5)
	IV	IV	Replace dependent variables	Bootstrap	Winsorize
	CHEI	CHEI	Total of meals eaten out	CHEI	CHEI
Income	2.7697***				
	(0.0028)				
Commuting time		−67.1698***	0.1255***	−1.0387***	−1.0387***
		(0.0000)	(0.0009)	(0.0001)	(0.0000)
Constant	71.2672***	68.7812***	68.6334***	61.5167***	−0.1083**
	(0.0000)	(0.0000)	(0.0000)	(0.0000)	(0.0179)
Control time effect	Yes	Yes	Yes	Yes	Yes
Control area effect	Yes	Yes	Yes	Yes	Yes
*N*	2,567	2,567	2,567	2,567	2,567
*R* ^2^	0.2837	0.0198	0.1490	0.2949	0.3009

*, ** and *** indicate significant at the 10, 5 and 1% levels, respectively. p-Values in parentheses.

Commuting time and dietary quality. Similarly, to address the endogeneity issue due to the bidirectional causality between commuting time and dietary quality, the present position selects the average annual price of commercial housing sales in each province as an instrumental variable (data from the National Statistical Yearbook https://data.stats.gov.cn/easyquery.htm?cn=E0103) and uses an instrumental variable 2sls regression to conduct endogeneity test. The average sales price of commercial real estate can reflect the population density and traffic pressure of a region, according to the “2023 Annual Traffic Analysis Report of China’s Major Cities” pointed out that the top 10 cities in the morning and evening peaks of the public transportation/car travel time for Shenzhen, Beijing, Guangzhou, Shanghai, Chengdu, etc., and the average sales price of commercial real estate in the region is basically convergent.

The regression results are shown in Table 7 (2), after solving the endogeneity problem, the commuting time is significantly negative at 1% level, indicating that the commuting time with can significantly reduce the quality of laborer’ diets.

#### 4.2.2 Robustness testing

To ensure the robustness of the empirical results. This study will replace the dependent variables and use Bootstrap sampling for robustness testing.

First, replacing dependent variables. Numerous studies have shown that eating out is associated with unhealthy diets (23, 24), so this study takes the number of eating out as a dependent variable for further testing, and the regression results are shown in Table 6 (1). There is a positive correlation between the increase in commuting time and the number of meals out. The result of “increased commuting time—increased number of meals away from home—decreased meal quality” further verified the robust negative relationship between commuting time and diet quality.

Second, bootstrap sampling test. Based on Bootstrap sampling method, the sample was sampled 1,000 times with put back and regressed, and the empirical results are shown in Table 7 (4). According to the empirical results, commuting time is still significantly negative at the 1% level, which again indicates the robustness of the previous findings.

Third, winsorizing the dependent variables. The CHEI variables in this study were calculated based on the grams of all food types consumed in 9 meals over 3 days in the CHNS database of the Dietary Survey of Residents, and the accuracy of the questionnaire data determines the precision of the CHEI variables. Given that there may be omissions in the questionnaire data collection process leading to the possible bias in the calculation of the variables, regression was conducted again after winsorizing the dependent variables by 1 and 99%. The regression results are shown in in Table 7 (5), and the commuting time variable is still significantly negative at the 1% level after the trimming, verifying the robustness of the previous findings.

#### 4.2.3 Mechanism analysis and nonlinearity test

Mechanism analysis. Table 8 reports the results of the regressions of commute time on specific food groups in the CHEI. Because the CHNS seasoning data are for food weighed at home, no regression analyses of seasonings are conducted in this section. The remaining foods were categorized according to the CHEI classification, which divides foods into five groups: grains and whole grains, vegetables, eggs and milk and nuts, fruits, and fish and seafood. As shown in Table 8 (1–5), commuting time was significantly negatively correlated with grains, vegetables, and egg and milk. As commuting time increased, intake of grains, vegetables, and egg and milk decreased, thus leading to structural changes in CHEI and a decrease in dietary quality scores. And the results showed that only 1,233 samples reported fruit intake, and the overall fruit intake of the sample was poor. Meanwhile, the increase in education level and income played a positive role in optimizing dietary structure.

**Table 8:** Regression results of mechanism analysis and nonlinearity test.

Variable	(1)	(2)	(3)	(4)	(5)	(6)
	Fish & meat & seafood	Vegetables	Egg & milk & nuts	Fruit	Total grains & whole grains	CHEI
Commuting time	−0.3239***	−0.2143**	−0.2637**	−0.2916	−0.0441	
	(0.0024)	(0.0152)	(0.0147)	(0.1226)	(0.6120)	
Commuting time^2^						−0.3190***
						(0.0002)
BMI	−0.0214	−0.0103	−0.0310	−0.0533*	−0.0014	−0.1072**
	(0.2673)	(0.5198)	(0.1120)	(0.0833)	(0.9296)	(0.0191)
Education level	0.1715***	−0.0042	0.2951***	0.0212	0.0870*	0.5287***
	(0.0024)	(0.9282)	(0.0000)	(0.8259)	(0.0580)	(0.0001)
Knowledge score	−0.0095	0.0154	0.0342**	0.0396	−0.0084	0.0337
	(0.5296)	(0.2149)	(0.0245)	(0.2350)	(0.4926)	(0.3443)
Marital status	−0.0030	−0.0255	0.0322	−0.1849	0.0688	0.2872
	(0.9768)	(0.7680)	(0.7613)	(0.3493)	(0.4206)	(0.2474)
Mall score	−0.0776***	0.0416*	−0.0062	0.0918*	0.1019***	0.0231
	(0.0051)	(0.0688)	(0.8234)	(0.0774)	(0.0000)	(0.7242)
Transportation score	0.0033	−0.0919***	0.0898**	0.1357*	−0.0729**	0.0050
	(0.9345)	(0.0058)	(0.0275)	(0.0818)	(0.0262)	(0.9583)
Income	0.1846*	−0.2471***	0.8697***	0.4982***	0.1739**	1.1827***
	(0.0812)	(0.0047)	(0.0000)	(0.0083)	(0.0437)	(0.0000)
Constant	6.0582***	13.9207***	1.4012***	2.4048	9.3158***	61.0131***
	(0.0000)	(0.0000)	(0.0000)	(0.0000)	(0.0000)	(0.0000)
Control time effect	Yes	Yes	Yes	Yes	Yes	Yes
Control area effect	Yes	Yes	Yes	Yes	Yes	Yes
*N*	2,567	2,567	2,567	1,233	2,567	2,567
*R* ^2^	0.1796	0.1039	0.2226	0.1301	0.1448	0.2940

*, ** and *** indicate significant at the 10, 5 and 1% levels, respectively. *p*-Values in parentheses.

Nonlinearity test. The quadratic term of commuting time is added to the model for regression to test the nonlinear effect of commuting time on dietary quality, and the empirical results are shown in Table 8 (6). According to the reality of the empirical results, the coefficient of the quadratic term of commuting time is negative at 1% level of significance, which indicates that the dietary quality of laborers will be further reduced with the increase of commuting time.

### 4.3 Heterogeneity analysis

#### 4.3.1 Heterogeneity analysis of commuting time

Table 9 reports the effects of different commute times on the quality of laborers’ diets. To test the effect of commuting time on laborers’ dietary quality, this study conducts further subgroup regressions. According to the definition of extreme commuting in the 2023 China Major Cities Commuting Monitoring Report, a one-way commute longer than 60 min is an extreme commute.

**Table 9:** Heterogeneity regression results of commuting time.

Variable	(1)	(2)	(3)
	Hours<0.6 CHEI	0.6 < = hours <1 CHEI	Hours > = 1.1 CHEI
Commuting time	−2.3185	−4.9474**	−1.5343***
	(0.1207)	(0.0367)	(0.0352)
BMI	−0.1402**	0.0101	−0.1672
	(0.0155)	(0.9112)	(0.2138)
Education level	0.3138*	1.0319***	0.7560**
	(0.0587)	(0.0003)	(0.0399)
Knowledge score	0.0763*	−0.0785	0.1021
	(0.0970)	(0.2810)	(0.2814)
Marital status	0.1253	0.4983	0.9524
	(0.6925)	(0.2943)	(0.2058)
Mall score	0.0650	−0.0919	−0.0498
	(0.4340)	(0.5196)	(0.7687)
Transportation score	0.1475	−0.2546	−0.1912
	(0.2159)	(0.2376)	(0.4509)
Income	0.8384***	0.8881	2.6244***
	(0.0076)	(0.1017)	(0.00000)
Constant	64.1304***	45.2367***	49.2643***
	(0.0000)	(0.0000)	(0.0000)
Control time effect	Yes	Yes	Yes
Control area effect	Yes	Yes	Yes
*N*	1574	582	412
*R* ^2^	0.2678	0.3016	0.4046

*, ** and *** indicate significant at the 10, 5 and 1% levels, respectively. *p*-Values in parentheses.

We use this criterion and the average commuting time (0.6 h) of the 6,973 samples for grouping. As shown in the table, commuting time is not significantly related to the regression results of dietary quality under 0.6 h. When the commuting time exceeds the average time and enters the extreme commuting situation, the commuting time is significantly negatively correlated with meal quality.

#### 4.3.2 Heterogeneity analysis of income

Table 10 reports the effect of commuting time on the dietary quality of laborers at different incomes. The regression results show that when per capita household income is at a low level, commuting time negatively affects the dietary health of laborers. This negative effect decreases as per capita household income increases. When per capita household income is higher than 1808 (ln 7.5), the negative impact of commuting time disappears.

**Table 10:** Heterogeneity regression results of income.

Variable	(1)	(2)	(3)
	Low income ≤6.5 CHEI	Medium 6.5< income <7.5 CHEI	High income ≥7.5 CHEI
Commuting time	−2.5448***	−1.2031***	0.4959
	(0.0000)	(0.0023)	(0.2181)
BMI	−0.0352	−0.2060***	−0.0958
	(0.6669)	(0.0058)	(0.1353)
Education level	0.5522**	0.6783***	0.7900***
	(0.0182)	(0.0004)	(0.0001)
Knowledge score	−0.0177	−0.0095	0.2028***
	(0.7925)	(0.8485)	(0.0010)
Marital status	0.9033**	0.2484	−0.3035
	(0.0371)	(0.5452)	(0.4320)
Mall score	0.3544***	0.0028	−0.1102
	(0.0013)	(0.9769)	(0.2790)
Transportation score	0.1638	−0.3184**	0.2653
	(0.2669)	(0.0262)	(0.1391)
Constant	62.3007***	−11.8758***	61.0587***
	(0.0000)	(0.0000)	(0.0000)
Control time effect	Yes	Yes	Yes
Control area effect	Yes	Yes	Yes
*N*	742	1111	714
*R* ^2^	0.3286	0.2709	0.1939

*, ** and *** indicate significant at the 10, 5 and 1% levels, respectively. *p*-Values in parentheses.

#### 4.3.3 Heterogeneity analysis of age

Table 11 reports the effect of commuting time on dietary quality of laborers of different ages. There are differences in the physical functions of people in different age groups, and people enter the aging stage in middle age, when the functions of the tissues and organs of the body begin to decline gradually. Usually, from the age of 30, the physiological functions of human beings decline at a rate of 0.7 to 1% per year (25, 26). Based on this, this paper is further divided into four groups according to age based on the total sample, (1): 29 years old and below, (2): 30–39 years old, (3): 40–49 years old, and (4): 50 years old and above, the specific regression results are shown in Table 9.

**Table 11:** Heterogeneity regression results of age.

Variable	(1)	(2)	(3)	(4)
	age<=29 CHEI	30 < =age <39 CHEI	40 < age <49 CHEI	age > = 50 CHEI
Commuting time	−1.0024	−0.8293*	−1.3520***	−1.4129***
	(0.2379)	(0.0767)	(0.0025)	(0.0032)
BMI	−0.0684	−0.0492	−0.2026***	−0.0779
	(0.6891)	(0.6220)	(0.0077)	(0.3280)
Education level	1.7067***	0.7218***	0.0107	0.5367***
	(0.0033)	(0.0090)	(0.9639)	(0.0325)
Knowledge score	0.271441**	0.0181	0.0379	−0.0240
	(0.0458)	(0.8317)	(0.4975)	(0.6992)
Marital status	0.4198	0.5436	−0.6599	0.7750
	(0.5250)	(0.2146)	(0.1487)	(0.1971)
Mall score	−0.0431	−0.1353	0.0980	0.1673
	(0.8492)	(0.2897)	(0.3787)	(0.1807)
Transportation score	0.1316	0.2466	−0.1874	−0.1160
	(0.6788)	(0.1519)	(0.2583)	(0.5228)
Income	2.4206**	0.1589	0.9790**	2.2217***
	(0.0162)	(0.7583)	(0.0192)	(0.0000)
Constant	49.2218***	69.3132***	77.1288***	67.3054***
	(0.0000)	(0.0000)	(0.0000)	(0.0000)
Control time effect	Yes	Yes	Yes	Yes
Control area effect	Yes	Yes	Yes	Yes
*N*	264	699	915	689
*R* ^2^	0.2632	0.3097	0.3054	0.2738

*, ** and *** indicate significant at the 10, 5 and 1% levels, respectively. *p*-Values in parentheses.

The effect of commuting time on dietary quality was not significant in the youth group during the period of perfect physical functioning, and with increasing age and gradual decline in physical functioning, there was a significant negative correlation between commuting time and dietary quality in group (2) (3) (4). This indicates that as age increases, the decline in dietary quality of the laborer caused by the increase in commuting time gradually increases, further confirming the negative effect of commuting time on diet. And it is necessary to optimize the commuting time and dietary intervention for the laborer at an early stage.

#### 4.3.4 Heterogeneity analysis of dining locations

Table 12 reports the effect of commuting time on the quality of laborers’ diet in different locations. The statistical results based on 9 meals in 3 days and 24 h for 2,567 samples show that home, company cafeteria, and restaurant are the main locations for meals. By calculating the frequency of the three locations in the 9 meals, this study will further conduct regression analysis on the dietary quality of laborers based on different locations. As the results are shown in Table 10, the increase of meal frequency in company cafeteria can significantly improve the dietary quality of the commuter group, and the increase of meal frequency in restaurant can significantly reduce the dietary quality of the commuter group. Further validation of the paper H2.

**Table 12:** Heterogeneity regression results of dining locations.

Variable	(1)	(2)	(3)
	CHEI	CHEI	CHEI
Meals at home	0.0924		
	(0.2442)		
Meals at corporation		0.2446**	
		(0.0473)	
Meals at restaurant			−0.3731***
			(0.0009)
BMI	−0.1063**	−0.1077**	−0.1030**
	(0.0205)	(0.0188)	(0.0244)
Education level	0.5444***	0.5002***	0.5610***
	(0.0001)	(0.0002)	(0.0000)
Knowledge score	0.0377	0.0352	0.0374
	(0.2918)	(0.3240)	(0.2950)
Marital status	0.3048	0.3147	0.3176
	(0.2206)	(0.2058)	(0.2010)
Mall score	0.0317	0.0227	0.0347
	(0.6305)	(0.7296)	(0.5965)
Transportation score	−0.0037	−0.0006	0.0016
	(0.9694)	(0.9954)	(0.9870)
Income	1.2026***	1.1768***	1.2468***
	(0.0000)	(0.0000)	(0.0000)
Constant	59.3344***	60.2017***	59.8495***
	(0.0000)	(0.0000)	(0.0000)
Control time effect	Yes	Yes	Yes
Control area effect	Yes	Yes	Yes
*N*	2,567	2,567	2,567
*R* ^2^	0.2906	0.2913	0.2933

*, ** and *** indicate significant at the 10, 5 and 1% levels, respectively. *p*-Values in parentheses.

### 4.4 Further analysis of family benefits

Table 13 reports the impact of commuting time on household members’ dietary quality. Whether this time squeeze poses a threat to the dietary health of household members is discussed further in this study. As shown in Table 13, commuting time not only reduces the dietary quality of the commuters themselves, but also reduces the dietary quality of the children in the household, posing a threat to family welfare. As shown by the regression coefficients in Table 13 (1) and (2), the coefficient of the children on the regression is −1.87 and the coefficient of myself is −1.03. The enhancement of commuting time has a greater impact on the children in the family on the quality of their diets than the reduction of the quality of their own diets.

**Table 13:** Heterogeneity regression results of family benefits.

Variable	(1)	(2)	(3)
	Sample CHEI	Sample’ children CHEI	Sample’ parents CHEI
Commuting time	−1.0387***	−1.8767**	−0.8316
	(0.0000)	(0.0304)	(0.1345)
BMI	−0.1083**	−0.3709**	−0.1499
	(0.0179)	(0.0277)	(0.1475)
Education level	0.5396***	−0.0460	0.6273**
	(0.0001)	(0.9224)	(0.0285)
Knowledge score	0.0356	−0.0495	0.0632
	(0.3181)	(0.6328)	(0.4636)
Marital status	0.2659	1.5683	0.5972*
	(0.2842)	(0.2044)	(0.0976)
Mall score	0.0230	0.8143***	−0.1090
	(0.7256)	(0.0003)	(0.4267)
Transportation score	0.0048	−0.1915	0.2409
	(0.9595)	(0.5170)	(0.2603)
Income	1.1755***	0.9343	1.5960***
	(0.0000)	(0.2790)	(0.0097)
Constant	61.5167***	69.8776***	57.3631***
	(0.0000)	(0.0000)	(0.0000)
Control time effect	Yes	Yes	Yes
Control area effect	Yes	Yes	Yes
*N*	2,567	338	471
*R* ^2^	0.2949	0.3210	0.3024

*, ** and *** indicate significant at the 10, 5 and 1% levels, respectively. *p*-Values in parentheses.

### 4.5 Further analysis of gender

Table 14 reports the impact of commuting time on different gender’ dietary quality. There may be differences in physical structure, work intensity, and family division of labor between males and females. Although there have been changes in recent years, in China, more women still undertake family caregiving responsibilities. Therefore, gender differences may lead to distinct outcomes. According to regression results, commuting time shows no significant impact on men’s dietary quality, while for women, it exhibits a statistically significant negative effect at the 1% level. This suggests that when commuting time increases, women are more likely to be adversely affected.

**Table 14:** Heterogeneity regression results of gender.

Variable	(1)	(2)
	Man CHEI	Woman CHEI
Commuting time	−0.6038	−1.3595***
	(0.1045)	(0.0001)
BMI	−0.0611	−0.0753
	(0.3839)	(0.2258)
Education level	0.4235**	0.6458***
	(0.0362)	(0.0003)
Knowledge score	0.0171	0.0512
	(0.7286)	(0.3219)
Marital status	0.2263	0.2067
	(0.5592)	(0.5236)
Mall score	−0.0205	0.0635
	(0.8367)	(0.4649)
Transportation score	0.0037	0.0127
	(0.9793)	(0.9216)
Income	1.2558***	1.1671***
	(0.0013)	(0.0004)
Constant	59.6865***	60.7023***
	(0.0000)	(0.0000)
Control time effect	Yes	Yes
Control area effect	Yes	Yes
*N*	1166	1401
*R* ^2^	0.2642	0.3180

*, ** and *** indicate significant at the 10, 5 and 1% levels, respectively. *p*-Values in parentheses.

## 5 Discussion

Consistent with the results of previous studies, the dietary quality of laborers in China is currently at a low level (27) and exhibits regional heterogeneity (28). The low quality of laborers’ diet in China mainly results from insufficient intake of meat, eggs, milk, vegetables, and fruits. Therefore, improving laborers’ dietary quality should involve increasing consumption of meat, eggs, milk, vegetables, and fruits. This contrasts with findings from some studies in developed countries, which indicate that increased consumption of meat and dairy products—along with higher intake of total and saturated fats—does not enhance diet quality (29–33). The likely explanation may be China’s status as a developing country where baseline dietary quality remains low for most of the population. Data from the CHEI’s 17 food groups reveal that most Chinese laborers fail to meet recommended intake standards for meat, eggs, and milk, indicating persistently low consumption levels.

There is broad consensus that poor dietary quality generally correlates with adverse health outcomes. The latest Dietary Guidelines for Americans (DGA) further emphasize the significant relationship between dietary patterns and human health, highlighting how establishing healthy eating patterns early in life and maintaining them thereafter may minimize risks of diet-related chronic diseases (34). A balanced diet plays a crucial role in sustaining physiological functions, while proactive dietary interventions have demonstrated preventive and therapeutic effects for certain conditions (35), showing particular promise in regulating metabolism, disease progression, and treatment responses (36, 37). Currently, dietary interventions have become a common and effective adjunct strategy in healthcare (38). The Chinese government prioritizes citizens’ nutrition, implementing the “Healthy China” strategy to enhance population dietary quality.

How to improve laborers’ dietary quality? Current research primarily adopts the “economic man” perspective regarding laborers (12–14). The economic man hypothesis posits that humans behave rationally in economic activities, with goal-oriented thinking and actions aimed at maximizing material utility. From this rational economic perspective, income growth increases food consumption, alters dietary structures, and alleviates malnutrition (39, 40). This aligns with our finding that dietary quality improves with income. However, prior literature has overlooked a critical factor: time constraints. Our results demonstrate that when laborers’ income reaches higher levels (per capita household income >1800), they become resilient to the negative dietary impacts of commuting time.

Prochaska and Schrimper (41), in their analysis of U.S. household consumption (1951–1970), first proposed dietary “time effects,” suggesting that income effect estimates for eating-out expenditures become biased when ignoring time constraints. This concurs with our findings; neglecting time effects leads to underestimating income’s impact on dietary quality. Regarding commuting time’s effect on physical health, Sun et al. (42) found that among Chinese urban residents, commuting time significantly reduces self-assessed health. Xiao et al. (43) similarly demonstrated that traffic-extended commutes increase negative emotions and decrease self-rated health. More detailed studies indicate that prolonged commuting elevates laborers’ BMI, increasing obesity risks (44).

However, the mechanism linking commuting time to health remains unclear. Our study further analyzes this relationship through dietary patterns, revealing that longer commuting time corresponds with lower dietary quality among laborers. Previous literature suggests that fast-paced modern lifestyles increase laborers’ preference for restaurant meals, particularly among overworked groups and youth lacking time for home cooking (45–47). Zhang et al. (48), surveying 6,099 employees (≤40 years) across China’s CDC system, showed that eating out negatively correlates with daily vegetable and fruit intake (49). This aligns with our findings: increased commuting time promotes restaurant dining, and among China’s three primary food venues (home, company cafeteria, restaurants), restaurant consumption reduces dietary quality—particularly for fruits, vegetables, and meat.

Notably, the study identifies older adults individuals, children, and women as dietary quality vulnerable groups. This implies that increased commuting time exacerbates negative dietary outcomes for these populations. Consequently, governments must incorporate targeted considerations for these groups when formulating dietary guidelines.

## 6 Conclusion

This study uses data from the 2004–2011 Chinese Household Nutrition and Health Survey (CHNS) to analyze the relationship between commuting time and residents’ dietary quality. The main conclusions of the study are as follows:

(1) An increase in income favors an increase in the quality of laborers’ diets. (2) Increased commuting time decreases the quality of the laborer’s diet, as reflected in decreased intake of grains, vegetables, eggs, and milk. (3) The longer the duration of the commute, the lower the quality of the laborer’s diet. However, a commuting time of less than 0.6 h does not affect the quality of laborers’ diets. (4) Increased commuting time has the greatest impact on the dietary health of children in the household.

## 7 Recommendations

Based on the above findings, this paper puts forward the following policy recommendations:

(1) Popularize dietary knowledge. The government should pay more attention to the dietary health of workers aged 25–60, as labor health is not only related to the accumulation of healthy human capital, but also to people’s well-being. The government should formulate reasonable dietary standards, popularize dietary knowledge, and guide residents to form scientific dietary habits.
(2) Optimize the urban layout. The Government should scientifically co-ordinate the layout of land for employment, housing, services, and transportation, to provide the public with more job opportunities close to their places of residence and reduce long-distance commuting. At the same time, it should improve the construction of urban rapid transit, increase investment in the construction of high-capacity public transportation such as rail transit and bus rapid transit systems, build a “30-min” urban transportation net, and unclog the urban transportation.
(3) Improve the availability of work meals. The potential of the workplace to improve the health of the workforce should be fully utilized. As a “health promotion priority” recognized by the World Health Organization (WHO) and the U. S. Centers for Disease Control and Prevention (CDC), workplaces should be actively utilized to enhance the health of the workforce, promote the construction of centralized employee cafeterias, optimize the dining environment of the workforce, and enhance the convenience of access to nutritious work meals. It should also actively guide neighboring catering organizations to provide safe, convenient, and nutritious group meal delivery, to provide reliable food consumption choices for the commuting population.

## 8 Limitation

This study still has some limitations in data acquisition. (1) The CHNS 3-day, 24-h diet data is not an average of the four seasons. But fortunately, the CHNS data were collected in the fall, a time when differences in food availability are minimal. (2) Unfortunately, car commuting is not recorded in the CHNS questionnaire, but this paper has used sufficient empirical analysis to demonstrate the relationship between commuting time and diet quality, and the existing results are credible. (3)This study focused on the impact of commuting time on the dietary quality of workers, but the existing data could not further examine the relationship between commuting time and poor health, and future research data will be further explored. (4)Shift work, care responsibilities or the variability of food access and other factors may also have an impact on the dietary health of workers, but due to data limitations, this part cannot be controlled.

## Data Availability

The datasets presented in this study can be found in online repositories. The names of the repository/repositories and accession number(s) can be found below: Data source: Statistical analysis based on data from China Health and Nutrition Survey in 2004, 2006, 2009 and 2011. https://www.cpc.unc.edu/projects/china.
